# Blood biomarker profiles in young-onset neurocognitive disorders: A cohort study

**DOI:** 10.1177/00048674241312805

**Published:** 2025-01-17

**Authors:** Oneil G Bhalala, Jessica Beamish, Dhamidhu Eratne, Patrick Summerell, Tenielle Porter, Simon M Laws, Matthew JY Kang, Aamira J Huq, Wei-Hsuan Chiu, Claire Cadwallader, Mark Walterfang, Sarah Farrand, Andrew H Evans, Wendy Kelso, Leonid Churilov, Rosie Watson, Nawaf Yassi, Dennis Velakoulis, Samantha M Loi

**Affiliations:** 1 Department of Medicine, Royal Melbourne Hospital, The University of Melbourne, Parkville, VIC, Australia; 2 Population Health and Immunity Division, Walter and Eliza Hall Institute of Medical Research, Parkville, VIC, Australia; 3 Neuropsychiatry Centre, The Royal Melbourne Hospital, Parkville, VIC, Australia; 4 Department of Psychiatry, The University of Melbourne, Parkville, VIC, Australia; 5 Centre for Precision Health, Edith Cowan University, Joondalup, WA, Australia; 6 Memory and Aging Center, UCSF Weill Institute for Neurosciences, University of California, San Francisco, San Francisco, CA, USA; 7 The Florey Institute of Neuroscience and Mental Health, Parkville, VIC, Australia; 8 School of Medical and Health Sciences, Edith Cowan University, Joondalup, WA, Australia

**Keywords:** Young-onset dementia, apolipoprotein E, polygenic risk scores, neurofilament light, glial fibrillary acidic protein, phosphorylated tau 181, primary psychiatric disorder, neurodegeneration, Alzheimer’s disease, multi-omic analyses

## Abstract

**Introduction:**

Young-onset neurocognitive symptoms result from a heterogeneous group of neurological and psychiatric disorders which present a diagnostic challenge. To identify such factors, we analysed the Biomarkers in Younger-Onset Neurocognitive Disorders cohort, a study of individuals <65 years old presenting with neurocognitive symptoms for a diagnosis and who have undergone cognitive and biomarker analyses.

**Methods:**

Sixty-five participants (median age at assessment of 56 years, 45% female) were recruited during their index presentation to the Royal Melbourne Hospital Neuropsychiatry Centre, a tertiary specialist service in Melbourne, Australia, and categorized as either early-onset Alzheimer’s disease (*n* = 18), non-Alzheimer’s disease neurodegeneration (*n* = 23) or primary psychiatric disorders (*n* = 24). Levels of neurofilament light chain, glial fibrillary acidic protein and phosphorylated-tau 181, apolipoprotein E genotype and late-onset Alzheimer’s disease polygenic risk scores were determined. Information-theoretic model selection identified discriminatory factors.

**Results:**

Neurofilament light chain, glial fibrillary acidic protein and phosphorylated-tau 181 levels were elevated in early-onset Alzheimer’s disease compared with other diagnostic categories. A multi-omic model selection identified that a combination of cognitive and blood biomarkers, but not the polygenic risk score, discriminated between early-onset Alzheimer’s disease and primary psychiatric disorders (area under the curve ⩾ 0.975, 95% confidence interval: 0.825–1.000). Phosphorylated-tau 181 alone significantly discriminated between early-onset Alzheimer’s disease and non-Alzheimer’s disease neurodegeneration causes (area under the curve = 0.950, 95% confidence interval: 0.877–1.00).

**Discussion:**

Discriminating between early-onset Alzheimer’s disease, non-Alzheimer’s disease neurodegeneration and primary psychiatric disorders causes of young-onset neurocognitive symptoms is possible by combining cognitive profiles with blood biomarkers. These results support utilizing blood biomarkers for the work-up of young-onset neurocognitive symptoms and highlight the need for the development of a young-onset Alzheimer’s disease-specific polygenic risk score.

## Background

Young-onset dementia, often defined as dementia diagnosed before the age of 65 years (Van de Veen et al., 2021), comprises a heterogeneous group of disorders that accounts for approximately 5% of all cases of dementia and have a prevalence of 119 per 100,000 individuals worldwide (Hendriks et al., 2021; Loi et al., 2023). Diagnosing young-onset dementia is challenging due to the atypical symptoms and cognitive profiles that can overlap with other causes of neurocognitive disorders in this age group, such as primary psychiatric disorders (PPD). Nearly one-third of individuals are misdiagnosed with PPD prior to the identification of a neurodegenerative cause and nearly 40% of individuals with young-onset neurocognitive symptoms have their initial diagnosis revised during follow-up, reflecting low diagnostic certainty with standard clinical work-up (Tsoukra et al., 2022; Woolley et al., 2011). Establishing a timely and accurate diagnosis is important to facilitate appropriate management and understand prognosis.

Blood biomarkers are emerging as powerful predictors of neurodegenerative conditions. Improvements in immunoassay technology, such as single molecule array (Simoa^®^), have yielded ultra-sensitive detection of protein biomarkers in blood, where concentrations are often in the pico- and femtomolar ranges (Mankhong et al., 2022). Neurofilament light chain (NfL) and glial fibrillary acidic protein (GFAP) are detected in blood samples from a variety of neurodegenerative conditions including late-onset Alzheimer’s disease, Lewy body disease and frontotemporal dementia (Bacioglu et al., 2016; Eratne et al., 2024a; Hansson et al., 2023) . Furthermore, hyperphosphorylated species of tau, such as those phosphorylated at threonine 181 (p-tau181), have demonstrated high specificity in distinguishing late-onset Alzheimer’s disease from other neurodegenerative conditions (Janelidze et al., 2020).

While different protein blood biomarkers reflect dynamic changes occurring in the setting of neurodegeneration, they vary in their ability to predict onset of disease prior to symptom onset. Contrastingly, genetics can be considered as capturing an individual’s static risk of developing neurodegeneration (Bhalala et al., 2024). Genetic variants have been identified using genome-wide association studies for conditions such as late-onset Alzheimer’s disease, Lewy body disease and Parkinson’s disease (Bellenguez et al., 2022; Chia et al., 2021; Kim et al., 2024; Kunkle et al., 2019). Polygenic risk scores have been derived from genome-wide association studies to calculate an individual’s risk of developing a particular neurodegenerative condition. For example, those with the highest 10% polygenic risk score values have nearly a 1.9-fold increase in the risk of late-onset Alzheimer’s disease compared with those in the lowest 10%, and this effect is additive to age and the apolipoprotein E (*APOE*) status, with the latter being the gene most strongly associated with late-onset Alzheimer’s disease (Bellenguez et al., 2022).

Leveraging multi-omic data by combining the static risk captured by genetic analyses such as the polygenic risk score, the dynamic risk identified by protein biomarkers, and standard clinical assessments can improve diagnostic accuracy for late-onset neurodegenerative conditions such as late-onset Alzheimer’s disease (Bhalala et al., 2024; Palmqvist et al., 2021; Ramanan et al., 2023). However, there is a paucity of data on how such an approach applies to diagnosing individuals with young-onset neurocognitive symptoms. Identifying clinical variables and blood biomarkers that are useful in discriminating between young-onset dementia causes may improve diagnostic accuracy and timeliness.

In this study, we sought to address these questions by analysing the Biomarkers in Younger-Onset Neurocognitive Disorders (BeYOND) cohort (Loi et al., 2020), a clinical cohort of individuals experiencing young-onset neurocognitive symptoms. In brief, BeYOND is a prospective observational cohort study evaluating clinical presentation, cognition, blood biomarkers and genetics. Recruitment criteria included individuals referred to the Neuropsychiatry Centre at the Royal Melbourne Hospital for a possible diagnosis of young-onset dementia who had psychiatric, behavioural, neurological and/or cognitive symptom onset prior to the age of 65 years. Participants were prospectively recruited during their index presentation. The study aimed to determine which combinations of clinical, cognitive, blood and genetic biomarker variables accurately differentiated causes of neurocognitive symptoms between the categories of early-onset AD (EOAD), non-AD neurodegeneration (nAD-ND) and PPD within this group (Supplemental File 1).

## Method

### Participant recruitment

The BeYOND study protocol has been described previously, with participant recruitment from June 2019 to December 2020 and a follow-up over 1 year (Loi et al., 2021a). For this study, we evaluated cognition, genetics and blood biomarkers with clinical characteristics cross-sectionally. This project was approved by the Melbourne Health Human Research Ethics Committee (MH 2018.371).

### Diagnosis

Diagnosis was made according to consensus criteria, including the McKhann criteria for AD (McKhann et al., 2011), the Movement Disorder Society diagnostic criteria for Parkinson’s disease (Postuma et al., 2015) and progressive supranuclear palsy (Höglinger et al., 2017), the 2017 McKeith criteria for dementia with Lewy bodies (McKeith et al., 2017) and the Rascovsky criteria for behavioural-variant frontotemporal dementia (Rascovsky et al., 2011). Niemann-Pick disease Type C, cerebral autosomal dominant arteriopathy with subcortical infarcts and leukoencephalopathy, vascular dementia and cerebellar degeneration were diagnosed based on a combination of clinical and imaging features, as well as genetic testing where available. Cerebrospinal fluid protein levels of amyloid-β and phosphorylated-tau at threonine 181 (p-tau181) were utilized for diagnosis of AD. Psychiatric diagnoses were made according to the Diagnostic Statistical Manual, Fifth Edition (American Psychiatric Association, 2013).

### Cognitive classification

Participants underwent formal neuropsychological assessments for the overarching domains of global cognition, memory (delayed recall) and executive function. Global cognitive function was determined using overall performance on the Neuropsychiatry Cognitive Assessment tool (Walterfang et al., 2006), which measures attention, memory, visuospatial, executive and language functions. A range of domain-specific neuropsychological tests were used to further assess memory and executive function, as shown in Supplementary Table 1.

Raw scores from the neuropsychological tests were converted into Z-scores based on normative data stratified by age and education as provided in the test manuals (Griffith et al., 2022, 2024). A Z-score was categorized into ‘impaired’ at 1.5 standard deviations below the normative score or ‘normal’ (not impaired’). If a participant completed more than one neuropsychological test in a particular domain, the classification for each test was determined. For differing classifications, two clinical neuropsychologists evaluated the data and formed a consensus opinion about the overall performance in that domain.

### Biomarker analyses

EDTA blood samples were collected from fasted participants and stored at −80°C. Plasma was tested in duplicates for selected biomarkers using Quanterix Simoa^®^ HD-X Neurology 2 Plex B for NfL and GFAP, and single Plex for p-tau181, according to the manufacturer’s specifications. ‘Age at assessment’ is the participant’s age at which blood samples were collected.

### APOE genotyping and polygenic risk score calculation

DNA was extracted from peripheral whole-blood samples and *APOE* genotype determined using TaqMan® genotyping assays (rs7412, assay ID: C____904973_10; rs429358, assay ID: C____3084793_20), (Life Technologies, Carlsbad, CA) on a QuantStudio 12K FlexTM Real-Time-PCR system (Applied Biosystems, Foster City, CA), using TaqMan® GTXpressTM Master Mix (Life Technologies) as per manufacturer instructions (Porter et al., 2018a, 2018b) Allele loads were quantified for ε2 and ε4 and values were assigned to each individual (0,1 or 2) per allele, representing their *APOE* ε2 and ε4 status.

Participant genetic data were derived from an Axiom™ Precision Medicine Diversity Array (Applied Biosystems™) and imputed using the Haplotype Reference Consortium panel, for greatest cross-over of single nucleotide polymorphisms (SNPs). Beta weights for polygenic risk score calculation and *p*-value thresholds for variant inclusion were sourced from the International Genomics of Alzheimer’s Project Alzheimer’s disease genome-wide association studies (Kunkle et al., 2019). Polygenic risk scores were calculated by first multiplying the number of effect alleles for each variant by the beta weights from the genome-wide association studies summary statistics, with the weighted variant scores summed for each individual. Polygenic risk scores were calculated at eight different genome-wide association studies *p-*value thresholds, from *p* < 5 × 10^-8^, reflecting the standard stringent genome-wide association studies-threshold, to *p* < 0.1, reflecting a suggestive-association threshold.

### Statistical analyses

R-package (version 4.3.1) (Team, 2023) was used for statistical analyses. Due to the potential bias that may arise with covariate-based imputations using relatively small sample sizes, missing values were not imputed. For non-parametric data, the Kruskal–Wallis test was used when comparing across three or more groups, with the Dunn test applied for post hoc analyses. For non-parametric data comparison across two groups, the Wilcoxon rank sum exact test was used. For categorical data, Fisher’s exact test, with pairwise post hoc analyses using the R package rcompanion (Mangiafico, 2023), was used. For other post hoc analyses, Benjamini–Hochberg correction, with false discovery rate of 0.05 was used. *p_nom_* represent un-adjusted (nominal) *p*-values and *p_adj_* represent adjusted *p*-values using post hoc analyses. Significance level α was set to 0.05.

For multinomial logistic regression, the R package nnet (Venables and Ripley, 2002) was used. Only participants with complete data (on variables analysed) were used for modelling. Multinomial logistic regression was performed using diagnostic categories as the dependent variable and age at assessment, sex and individually tested protein biomarker levels as the independent variables. *p_nom_* values were calculated using the Wald statistic.

The R package MuMIn (Bartoń, 2024) was used for information-theoretic model selection of general linear mixed models composed of all available participant variables. Only participants with complete data were analysed. DeLong test was used to compare resulting models.

Plots were made the R packages ggplot2, ggbreak and ggstatsplot (Patil, 2021).

## Results

### BeYOND participant demographics

In all, 72 participants were recruited, with 65 of them classified within the diagnostic categories of either EOAD (*n* = 18), nAD-ND (*n* = 23), or PPD (*n* = 24, Table 1). nAD-ND causes consisted of behavioural-variant frontotemporal dementia with definite frontotemporal lobar degeneration pathology (*n* = 6), probable behavioural-variant frontotemporal dementia (*n* = 4), Parkinson disease (*n* = 3), Niemann-Pick disease type C (*n* = 3), cerebellar degeneration (*n* = 2), cerebral autosomal dominant arteriopathy with subcortical infarcts and leukoencephalopathy (*n* = 1), progressive supranuclear palsy (*n* = 1), dementia with Lewy bodies (*n* = 1), vascular dementia (*n* = 1) and that of unclear etiology (*n* = 1). PPD causes consisted of depression (*n* = 7), schizophrenia (*n* = 4), bipolar affective disorder (*n* = 3), subjective cognitive impairment (*n* = 2), schizoaffective disorder (*n* = 2), obsessive compulsive disorder (*n* = 1), psychosis (*n* = 1), delusional disorder (*n* = 1), functional neurological disorder (*n* = 1) and ‘other specified mental disorder’ (*n* = 2). The EOAD, nAD-ND and PPD groups were similar for age of symptom onset, age at assessment, sex and *APOE* genotypes (Table 1). Missing data ranged between 0% and 26% (Table 1, Supplementary Table 2).

**Table 1.** BeYOND study demographics and *APOE* genotypes.

	Overall *N* = 65	EOAD *N* = 18	nAD-ND *N* = 23	PPD *N* = 24
Age of onset^a^	56 (50, 60)	55 (52, 57)	59 (48, 61)	56 (50, 57)
(Missing) *	*7* (*11%*)	*0*	*6* (*26%*)	*1* (*4.2%*)
Age at assessment^a^	58 (55, 62)	59 (55, 62)	61 (54, 64)	57 (54, 60)
Sex^b^				
Female	29 (45%)	9 (50%)	9 (39%)	11 (46%)
Male	36 (55%)	9 (50%)	14 (61%)	13 (54%)
*APOE* genotype^b^				
ε2/ε3	5 (8.8%)	1 (6.3%)	1 (5.3%)	3 (14%)
ε3/ε3	32 (56%)	8 (50%)	12 (63%)	12 (55%)
ε3/ε4	18 (32%)	7 (44%)	4 (21%)	7 (32%)
ε4/ε4	2 (3.5%)	0 (0%)	2 (11%)	0 (0%)
(Missing) *	*8* (*12%*)	*2* (*11%*)	*4* (*17%*)	*2* (*8.3%*)

*APOE*: apolipoprotein E; EOAD: early-onset Alzheimer’s disease; nAD-ND: non-AD neurodegeneration; PPD: primary psychiatric disorder.

If not indicated, there were no missing data for the variable.

a Median (interquartile range).

b *n* (%).

* Number (percentage) of participants with missing/unknown data for particular variable.

Of the seven individuals that were not classified as having one of the three diagnostic categories listed above, five had a diagnosis of mild cognitive impairment of unclear aetiology, one had autoimmune encephalitis and one had primary angiitis of the central nervous system. These seven individuals were excluded from further analyses as the primary aim of the study was to differentiate between the three aforementioned diagnostic categories.

### Cognitive profiles among diagnostic categories

Executive function was similar between the EOAD, nAD-ND and PPD diagnostic groups (*p_nom_* = 0.14) while global cognitive function and memory function were impaired in proportionally more EOAD than nAD-ND or PPD groups (Table 2 and Supplementary Figure 1). In particular, 100% of those with EOAD had global cognitive impairment, compared to 55% of those with PPD (*p_adj_* < 0.005). Similarly, memory was classified as impaired in 82% of those with EOAD, compared with 26% of those with nAD-ND and 46% of those with PPD (*p_adj_* < 0.005 for EOAD vs nAD-ND and *p_adj_* < 0.05 for EOAD vs PPD).

**Table 2.** Cognitive outcomes and protein blood biomarker levels by diagnostic category.

	EOAD	nAD-ND	PPD	*p*-value^a^
Global cognition				0.001
Impaired	18 (100%)	15 (83%)	12 (55%)	
Intact	0 (0%)	3 (17%)	10 (45%)	
Memory function				0.002
Impaired	14 (82%)	5 (26%)	11 (46%)	
Intact	3 (18%)	14 (74%)	13 (54%)	
Executive function				0.14
Impaired	13 (76%)	15 (75%)	12 (50%)	
Intact	4 (24%)	5 (25%)	12 (50%)	
Biomarker				*p*-value^b^
NfL	21 (17, 27)	15 (10, 33)	10 (9, 14)	<0.001
GFAP	198 (154, 262)	77 (53, 112)	56 (48, 101)	<0.001
p-tau181	3.33 (3.04, 3.87)	1.69 (1.22, 1.97)	1.15 (0.94, 1.52)	<0.001

EOAD: early-onset Alzheimer’s disease; nAD-ND: non-AD neurodegeneration; PPD: primary psychiatric disorder.

Values indicate number (%) or median (interquartile range).

a Fisher’s Exact test between diagnostic groups.

b Kruskal–Wallis rank sum test.

### Protein blood biomarker levels

**Figure 1.**
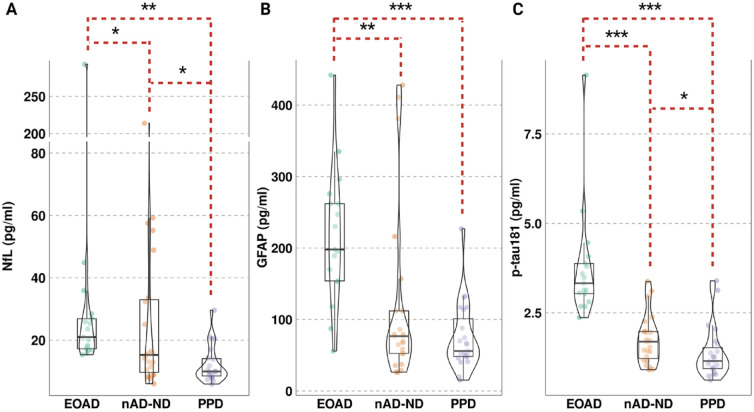
Blood biomarkers levels for NfL, GFAP and p-tau181 by diagnostic category. Violin plots of blood biomarker levels (pg/mL) for (A) neurofilament light chain (NfL), (B) glial fibrillary acidic protein (GFAP) and (C) phosphorylated-tau 181 (p-tau181) per diagnostic categories of early-onset Alzheimer’s disease (EOAD), non-AD neurodegeneration (nAD-ND) and primary psychiatric disorders (PPD). Boxplots represent the median and interquartile range. Adjusted (Dunn) *p*-value notation *p_adj_ < 0.05; **p_adj_ < 5 × 10^-3^; ***p_adj_ < 5 × 10^-5^.

Blood biomarkers levels for NfL, GFAP and p-tau181 were significantly different between the three categories, with levels higher in EOAD compared with the other categories (Figure 1, Table 2).

Median NfL levels were 1.4-fold higher in participants with EOAD (21 pg/ml) compared with nAD-ND (15 pg/mL, *p_adj_* = 0.02, Supplementary Table 3) and 2.1-fold higher compared with those with PPD (10 pg/mL, *p_adj_* = 5.6 x 10^-5^). Median NfL levels were also elevated in nAD-ND compared with PPD (1.5-fold, *p_adj_* = 0.02).

In those with EOAD, median GFAP levels were 3.5-fold higher (198 pg/mL) than in those with PPD (56 pg/mL, *p_adj_* = 1.3 × 10^-5^) and nearly 2.6-fold higher those with nAD-ND (77 pg/mL, 1.8-fold, *p_adj_* = 4.0 × 10^-4^). While median GFAP levels were over 1.3-fold higher in nAD-ND compared with PPD, statistical significance was not found (*p_adj_* = 0.14).

Median p-tau181 levels were nearly 2-fold higher in EOAD (3.33 pg/mL) compared with nAD-ND (1.69 pg/mL, *p_adj_* = 3.2 × 10^-5^) and 2.9-fold higher compared with PPD (1.15 pg/mL, *p_adj_* = 1.9 × 10^-8^). Similarly, nAD-ND had 1.5-fold higher median p-tau181 levels than PPD (*p_adj_* = 0.04). Pairwise comparisons between diagnoses with the adjusted (Dunn) *p*-values for the three protein blood biomarkers are provided in Supplementary Table 3.

We further tested the association between protein blood biomarkers and diagnostic categories, adjusted for age at assessment and sex. Participants without missing data for these variables were analysed using multinomial logistic regression models. Demographic variables between the diagnostic categories of participants analysed in these models were similar (Supplementary Table 4).

Compared with PPD, NfL demonstrated an odds ratio (OR) = 1.16 [95% CI: 1.05–1.29] for EOAD (*p_adj_* = 0.01) and OR = 1.16 [1.04–1.28] for nAD-ND (*p_adj_* = 0.01, Supplementary Table 5). Comparing between EOAD and nAD-ND, OR = 1.00 ([0.99–1.02], *p_adj_* = 0.61). While the OR for GFAP was statistically significant (*p_adj_* < 0.05) for EOAD vs PPD, the magnitude of the effect was quite small with OR = 1.02. Contrastingly, p-tau181 analyses yielded a large OR of 41.26 ([5.68–301.57], *p_adj_* = 0.002) and of 16.95 ([2.79–104.00], *p_adj_* = 0.006) for EOAD vs PPD and EOAD vs nAD-ND, respectively.

### Polygenic risk score associations

There were no statistically significant associations between polygenic risk score values, calculated using eight different late-onset Alzheimer’s disease genome-wide association studies (Kunkle et al., 2019) *p-*value thresholds and diagnostic categories (Supplementary Figure 2, Supplementary Tables 6 and 7). There was a trend for association using the stringent genome-wide association studies *p-*value threshold of 5 × 10^-8^ to calculate the polygenic risk score, with a median polygenic risk score value of 5.50 (interquartile range [IQR]: 4.14–6.14) in those with EOAD compared with 3.91 (IQR: 2.68–6.89) in those with nAD-ND and 2.45 (IQR: 0.70–4.69) in those with PPD (*p_nom_* = 0.085 for group comparison, Supplementary Figure 3).

#### Information-theoretic model selection identifies key variables for pairwise discrimination between diagnostic categories

To determine which combinations of clinical and biomarker variables best discriminated between diagnostic categories, we utilized an information-theoretic approach for model selection. Models with the lowest Akaike information criterion (AIC) with correction for finite sample sizes were selected as they represented the optimal trade-off between model fit and model complexity (Bartoń, 2024; Palmqvist et al., 2021). Variables included in the model selection were age at symptom onset, age at assessment, sex, cognitive function (global, memory and executive), blood biomarker levels (NfL, GFAP and p-tau181), *APOE* ε2 and ε4 allele status and the polygenic risk score (using genome-wide association studies *p*-value threshold of 5 × 10^-8^). Due to the model structure, only individuals with complete data could be analysed, yielding 46 participants across the three diagnostic categories with similar demographic variables among them (Supplementary Tables 8–10).

For discriminating between EOAD and PPD within the BeYOND cohort, the best model (Model 1A, as denoted by producing the lowest AIC) contained five variables: *APOE* ε2 and ε4 status, global cognitive function, NfL and p-tau181 (Supplementary Table 11); p-tau181 had the largest odds ratio of the included variables in Model 1, with an OR = 1.48 [95% CI: 1.33–1.65]. Interestingly, *APOE* ε2 and ε4 status both had OR < 1 for association with EOAD, compared with PPD. Three parsimonious models (Models 2A–4A) equivalent to Model 1A (as denoted by having a ΔAIC < 2 compared with Model 1A) were identified. The area under the receiver operating curves (AUCs) for Models 1A–4A were very high (Supplementary Figure 4A), ranging from 0.975 to 1.000 and were not statistically different (DeLong *p_nom_* > 0.05, Supplementary Table 11). We next tested how models containing only one of the variables identified in Model 1A performed. Models 5A–7A and 9A were inferior to Models 1A–4A with respect to both ΔAIC and AUC values. Model 8A, containing only the p-tau181 variable, demonstrated a high AUC of 0.954 [95% CI: 0.887–1.000], but with a ΔAIC > 2, indicated an inferior model compared with Models 1A–4A. Assessing all models analysed for information-theoretic model selection revealed that p-tau181 had a sum of weights (SoW) = 1.00 (indicating this variable was present in all models), while only NfL, *APOE* ε2 and global cognitive function had a SoW ⩾ 0.5 (indicating the importance of these variables as they were present in most of the well supported models based on AIC, Supplementary Figure 5).

To discriminate EOAD from nAD-ND, the best model (Model 1B) required only the variable p-tau181 with an AUC of 0.950 [95% CI: 0.877–1.000] and an OR = 1.63 [95% CI: 1.38–1.93] (Supplementary Table 12, Supplementary Figure 4B); p-tau181 had a SoW = 1 (Supplementary Figure 5). No other variable had a SoW ⩾ 0.5, indicating that no other variables were considered significant for this comparison. In determining which variables would discriminate between all neurodegenerative cases (combining EOAD and nAD-ND) and PPD, the best model (Model 1C) contained seven variables: age at assessment, *APOE* ε2 and ε4 status, global cognitive function, polygenic risk score, p-tau181 and sex (Supplementary Table 13, Supplementary Figure 4C), yielding an AUC = 0.984 [95% CI: 0.960–1.000]. Interestingly, global cognitive function had the largest magnitude of effect size with OR of 0.56 (95% CI: 0.43–0.71), followed by *APOE* ε2 (OR = 0.69), sex (OR = 1.37) and p-tau181 (OR = 1.28). One parsimonious model (Model 2C) contained six variables (polygenic risk score was not included) and demonstrated a similar AUC (0.975, 95% CI: 0.940–1.00, DeLong *p-*value = 0.35). Models 3C–9C, each containing only one variable from Model 1C, were all inferior to Models 1C and 2C with ΔAIC > 19. SoW for p-tau181 and global cognitive function was 1.00 and 0.99, respectively (Supplementary Figure 5). Other variables with SoW ⩾ 0.5 included sex (0.91), age at assessment (0.73), *APOE* ε2 (0.71), polygenic risk score (0.58), *APOE* ε4 (0.52) and memory function (0.50), indicating that more variables may contribute to discriminating between neurodegeneration and PPD compared with between EOAD vs PPD and EOAD vs nAD-ND.

## Discussion

We determined how clinical, cognitive, protein blood biomarker and genetic variables differentiated causes of neurocognitive symptoms into the categories of EOAD, nAD-ND and PPD within the BeYOND cohort. We found that levels of p-tau181 were higher in EOAD compared with nAD-ND and PPD when accounting for the covariates of age at assessment and sex. The association of the polygenic risk score with diagnostic categories did not reach statistical significance, but was suggestive. A model containing global cognitive function and levels of p-tau181 and NfL significantly discriminated between EOAD and PPD, while a model containing only p-tau181 significantly discriminated between EOAD and nAD-ND causes.

Blood biomarkers can differentiate and diagnose causes of late-onset cognitive impairment, especially late-onset Alzheimer’s disease (Chouliaras et al., 2022; Hansson et al., 2023; Qu et al., 2021; Saunders et al., 2023). In particular, NfL has been implicated in a wide range of neurodegenerative conditions (Bacioglu et al., 2016), including late-onset Alzheimer’s disease (Mattsson et al., 2017), frontotemporal dementia (Rohrer et al., 2016) and amyotrophic lateral sclerosis (Gaiani et al., 2017). This protein biomarker has shown utility in distinguishing neurodegenerative conditions from PPD (Eratne et al., 2022, 2024a). In our cohort, we found that NfL could function similarly, with EOAD demonstrating higher blood levels than nAD-ND and PPD. NfL continued to differentiate between neurodegenerative and PPD cases when adjusted for age at assessment and sex, factors which can influence protein blood biomarker levels (Eratne et al., 2024a; Sarto et al., 2024; Simrén et al., 2022; Tsiknia et al., 2022). In addition, blood-based NfL is found to be more sensitive in differentiating PPD and dementia in the younger age group (40–60 years), compared with 60–70 years (Eratne et al., 2024b; Light et al., 2024). The predictive power of NfL, however, was not demonstrated between EOAD and nAD-ND causes, supporting emerging data that NfL can be considered as a general marker of neurodegeneration (Ashton et al., 2021).

We found elevated GFAP levels in EOAD compared with nAD-ND and PPD. However, the adjusted odds ratio for GFAP (OR = 1.01–1.02) was low and of unclear clinical significance for EOAD vs PPD and vs nAD-ND. This differs from a prospective study of 110 individuals with EOAD and 50 controls, where blood GFAP levels demonstrated an AUC of 96% in discriminating between these two groups (Lv et al., 2023). A potential confounder in comparing these two studies is that the comparator group is individuals with PPD in our study, while healthy controls were used in the other, as well as the difference in samples sizes. Overall, our findings support the hypothesis that GFAP may be a marker in a number of neurodegenerative conditions (Abdelhak et al., 2022; Benussi et al., 2020; Cicognola et al., 2021; Oeckl et al., 2019). More work is needed to elucidate what blood GFAP levels add in discerning the cause of neurocognitive symptoms, especially in the younger populations.

A strength of this study is the identification of specific variables that contribute to diagnosis, as the diagnosis of neurodegenerative diseases is moving towards a multi-omics approach (Abdelhak et al., 2022). To the best of our knowledge, this is the first use of information-theoretic model selection (Bartoń, 2024) of multi-omic variables in a clinical cohort of young-onset neurocognitive disorders. A benefit of multi-model inference is the ability to determine important variables in a less biased manner as all studied variables can be included in these analyses, with the best model, as well as equivalent parsimonious models, identified. Using this approach, we found that global cognitive function and the blood biomarker levels of NfL and p-tau181 were sufficient to discriminate between EOAD and PPD. Similarly, p-tau181 alone was sufficient to discriminate EOAD from nAD-ND cases. Discriminating between all causes of neurodegeneration from PPD required more variables including *APOE* ε2 status, sex, p-tau181 levels, as well as global cognitive and memory function. These findings are similar to model selections performed in late-onset Alzheimer’s disease, where inclusion of p-tau181, *APOE* status, brain imaging and cognitive testing (memory and executive) were able to predict conversion to late-onset Alzheimer’s disease in individuals with subjective cognitive impairment or mild cognitive impairment (Palmqvist et al., 2021). Additional studies of individuals with young-onset neurocognitive symptoms may reveal divergent variables between early-onset and late-onset causes, though such studies will need to have consistent definitions of early-onset dementia (Reitz et al., 2020). These findings support further hypothesis testing that single blood biomarkers may be used in the appropriate context to risk stratify individuals for neurodegeneration, which may be useful in both clinical care and clinical trials (Hansson et al., 2022, 2023; Self and Holtzman, 2023).

Multiple studies have demonstrated that p-tau181 is a very specific biomarker for late-onset Alzheimer’s disease (Janelidze et al., 2020; Karikari et al., 2020; Thijssen et al., 2020). However, studies are lacking in describing the relationship between EOAD and p-tau181, especially in non-familial cases. One study did find elevated cerebrospinal fluid levels of p-tau181 in those with EOAD compared with healthy controls (Kaur et al., 2020). Our study extends this finding to blood, with plasma p-tau181 levels demonstrating a robust ability to discriminate EOAD from other causes of young-onset neurocognitive symptoms.

AD-based polygenic risk scores have been constructed using late-onset cases, which has shown strong ability to discriminate late-onset Alzheimer’s disease cases from healthy controls (Bellenguez et al., 2022). In our cohort, there was a trend towards a higher polygenic risk score in EOAD compared to nAD-ND and PPD when restricting the polygenic risk score. Overall, the transferability of a polygenic risk score derived from a late-onset Alzheimer’s disease population to an EOAD population is mixed, with some studies demonstrating poor correlation (Mantyh et al., 2023; Schott et al., 2016). Other studies have shown a potential role of a late-onset Alzheimer’s disease-derived polygenic risk score in predicting EOAD cases, though differences in age of participants and *APOE* status may confound direct comparisons (Cruchaga et al., 2018; de Rojas et al., 2021; Fulton-Howard et al., 2021; Huq et al., 2021). These findings indicate the need for an EOAD-derived polygenic risk score, especially in individuals with non-familial cases (Mol et al., 2022).

The relationship between *APOE* ε4 status and odds of being diagnosed with EOAD versus PPD was a surprising result, as the model selection suggested that a higher *APOE* ε4 allele count was associated with a higher probability of being diagnosed with PPD compared with EOAD. While *APOE* ε4 status is associated with a younger age-of-onset in late-onset Alzheimer’s disease and familial cases of AD, its role in sporadic cases of AD is less clear (Liu et al., 2013; Olarte et al., 2006). This study was not powered to detail the relationship between *APOE* ε4 status, age of onset and diagnosis. Further work is needed to elucidate this relationship and to determine if our findings are due to differences in the genetic contributions to EOAD or limitations due to sample size.

There are additional limitations to our study that warrant careful consideration. Analysis based on the relatively small size of 65 participants from the BeYOND cohort must be acknowledged. Diagnostic heterogeneity, as to the specific cause of young-onset neurocognitive symptoms, was very high and necessitated formation of three diagnostic categories to perform analyses. The magnitudes of effect sizes greatly depend on the relative proportions of the different diagnostic categories within the study cohort. We attempted to mitigate this issue by accounting for covariates and performing information-theoretic model selection, reducing bias and penalizing model over-fitting. However, these are also affected, to a certain degree, by the diagnostic proportions included in each model. Large external cohort studies, as well as meta-analyses, of young-onset neurocognitive symptoms are needed to validate our findings. With respect to biomarkers, we did not have access to standardized neuroimaging or emerging protein biomarkers like p-tau231, with the latter showing a differing role in late-onset Alzheimer’s disease diagnosis (Self and Holtzman, 2023). We also calculated polygenic risk scores using a genome-wide association studies derived from late-onset Alzheimer’s disease cases; (Kunkle et al., 2019) there are recent late-onset Alzheimer’s disease-polygenic risk score with more implicated genetic loci (Bellenguez et al., 2022; de Rojas et al., 2021). However, these recent studies use ‘proxy’ cases, where an individual who is asymptomatic is considered a genome-wide association studies case if they have at least one first degree relative diagnosed with late-onset Alzheimer’s disease, which may distort the relative contribution of genome-wide association studies loci to the risk of developing AD (Escott-Price and Hardy, 2022). It is unclear how proxy cases affect late-onset Alzheimer’s disease genome-wide association studies transferability in EOAD cases.

The BeYOND cohort studied here is similar to other studies in its distribution of diagnostic causes for young-onset neurocognitive symptoms, thereby adding to this field of research and supporting the generalizability of our results (Loi et al., 2021b; Rossor et al., 2010). Overall, this study adds to our understanding of the multi-omic variables aiding in the diagnosis of young-onset neurocognitive symptoms. Our findings support further research into the use of protein blood biomarkers and cognitive profiles in the diagnostic pathway as well as identify the need for the development and validation of an EOAD polygenic risk score.

## Supplemental Material

sj-docx-1-anp-10.1177_00048674241312805 – Supplemental material for Blood biomarker profiles in young-onset neurocognitive disorders: A cohort study Supplemental material, sj-docx-1-anp-10.1177_00048674241312805 for Blood biomarker profiles in young-onset neurocognitive disorders: A cohort study by Oneil G Bhalala, Jessica Beamish, Dhamidhu Eratne, Patrick Summerell, Tenielle Porter, Simon M Laws, Matthew JY Kang, Aamira J Huq, Wei-Hsuan Chiu, Claire Cadwallader, Mark Walterfang, Sarah Farrand, Andrew H Evans, Wendy Kelso, Leonid Churilov, Rosie Watson, Nawaf Yassi, Dennis Velakoulis and Samantha M Loi in Australian & New Zealand Journal of Psychiatry

sj-docx-2-anp-10.1177_00048674241312805 – Supplemental material for Blood biomarker profiles in young-onset neurocognitive disorders: A cohort study Supplemental material, sj-docx-2-anp-10.1177_00048674241312805 for Blood biomarker profiles in young-onset neurocognitive disorders: A cohort study by Oneil G Bhalala, Jessica Beamish, Dhamidhu Eratne, Patrick Summerell, Tenielle Porter, Simon M Laws, Matthew JY Kang, Aamira J Huq, Wei-Hsuan Chiu, Claire Cadwallader, Mark Walterfang, Sarah Farrand, Andrew H Evans, Wendy Kelso, Leonid Churilov, Rosie Watson, Nawaf Yassi, Dennis Velakoulis and Samantha M Loi in Australian & New Zealand Journal of Psychiatry

sj-xlsx-3-anp-10.1177_00048674241312805 – Supplemental material for Blood biomarker profiles in young-onset neurocognitive disorders: A cohort study Supplemental material, sj-xlsx-3-anp-10.1177_00048674241312805 for Blood biomarker profiles in young-onset neurocognitive disorders: A cohort study by Oneil G Bhalala, Jessica Beamish, Dhamidhu Eratne, Patrick Summerell, Tenielle Porter, Simon M Laws, Matthew JY Kang, Aamira J Huq, Wei-Hsuan Chiu, Claire Cadwallader, Mark Walterfang, Sarah Farrand, Andrew H Evans, Wendy Kelso, Leonid Churilov, Rosie Watson, Nawaf Yassi, Dennis Velakoulis and Samantha M Loi in Australian & New Zealand Journal of Psychiatry
